# Mantle Cell Lymphoma Causing Recurrent Pleural Effusions: A Case Report

**DOI:** 10.7759/cureus.48945

**Published:** 2023-11-17

**Authors:** Michael Obregon, Akshay Kohli, Mingchen Song

**Affiliations:** 1 Internal Medicine, Southern Illinois University School of Medicine, Springfield, USA; 2 Pulmonary and Critical Care Medicine, Southern Illinois University School of Medicine, Springfield, USA

**Keywords:** pleura, thoracentesis, pulmonary, pleural effusion, mantle cell lymphoma

## Abstract

Mantle cell lymphoma (MCL) is a rare type of B cell non-Hodgkin’s lymphoma. MCL is most commonly identified in the gastrointestinal tract. Yet, many other extranodal sites have been described in the literature, including the rare instances of the primary site being the pleura of the lung. We present a case with a 73-year-old female who presented with a three-month history of unintentional weight loss, nocturnal fever, and night sweats. She had recurrent left pleural effusions; however, thoracentesis and pleural fluid cytology were negative for malignancy. A definitive diagnosis was achieved after the patient underwent video-assisted thoracic surgery. MCL presenting as a pleural effusion is rarely reported in the literature.

## Introduction

Mantle cell lymphoma (MCL) is a subset of B cell non-Hodgkin’s lymphoma (NHL) first described in 1982 by Weisenburger et al. [1]. MCL is a rare malignancy with an estimated yearly incidence rate of 0.8 cases per 100,000 individuals in the United States and represents 3%-10% of adult-onset NHL. It is more common in males with a ratio of about 3:1 described in most studies. Incidence increases with age, and the median age at diagnosis is typically 68 years [2,3]. The majority of MCL is the result of overexpression of cyclin D1 secondary to chromosomal translocation of t(11;14) (q13;q32). A large heterogeneity exists in genetic aberrations associated with MCL, which results in variable clinical course and prognosis [4].

Clinical features include generalized lymphadenopathy along with bone marrow involvement. Patients can also have systemic symptoms such as fever, night sweats, and weight loss. MCL has been found in all the organ systems of the body, however, pleural involvement is exceedingly rare. Argatoff et al. [5] described one case of MCL out of 80 cases from 1998 to 1995 in British Columbia, that involved the pleura of the lung.

Diagnosis of MCL is usually made by excisional biopsy of the lymph node or an extra-nodal site. Peripheral blood flow cytometry can have CD 200 expression characteristic of MCL, and it is found to be positive for B-cell markers (CD19, CD20, CD22, CD79a). It is distinguished from other B-cell lymphomas by diffuse positivity for cyclin D1 and SOX11. SOX11 is usually negative in indolent MCL. Rare cases are negative for cyclin D1 but can still be positive for SOX11. MCL is typically negative for BCL6, CD10, and CD23 (while CLL is CD23 positive). Rare cases of MCL are CD10 positive. Testing for P53 mutation is recommended to help in the prognostication of MCL. Bone marrow biopsy should be considered depending on the presentation. Imaging studies, such as computed tomography, might be done initially to evaluate presenting symptoms or to identify involved lymph nodes and extra-nodal sites. FDG-PET/CT is recommended to look for tracer uptake and identify the extent of involvement. Previously, the median overall survival was three to five years, but with advances in therapy, prognosis has improved [1-6].

We present a case of a patient who presented with B symptoms and a unilateral pleural effusion. The patient underwent multiple thoracentesis procedures for recurrent pleural effusion, all cytology was negative. Ultimately the patient underwent video-assisted thoracoscopy surgery (VATS) and was found to have MCL of the pleura.

## Case presentation

A 73-year-old female with a past medical history of hypertension, hyperlipidemia, hypothyroidism, and asthma presented to the emergency department (ED) for acute onset palpitations and retrosternal chest pressure for one hour. Upon further evaluation, the patient reported progressively worsening dyspnea with exertion, nocturnal fever, night sweats, and unintentional weight loss of 40 lbs starting three months prior to presentation. The patient denied diarrhea, change in the caliber of her stools, melena, or hematochezia. On physical exam, decreased lung sounds in the left lower lung field were noted, and there was 2+ pitting edema to the tibial tuberosity bilaterally. Her past surgical history included tonsillectomy, right knee arthroscopy, and cesarean section. She denied a family history of malignancies. The patient smoked one pack per day for 20 years but quit smoking 37 years ago. She denied drinking alcohol or use of illicit drugs.

**Figure 1:**
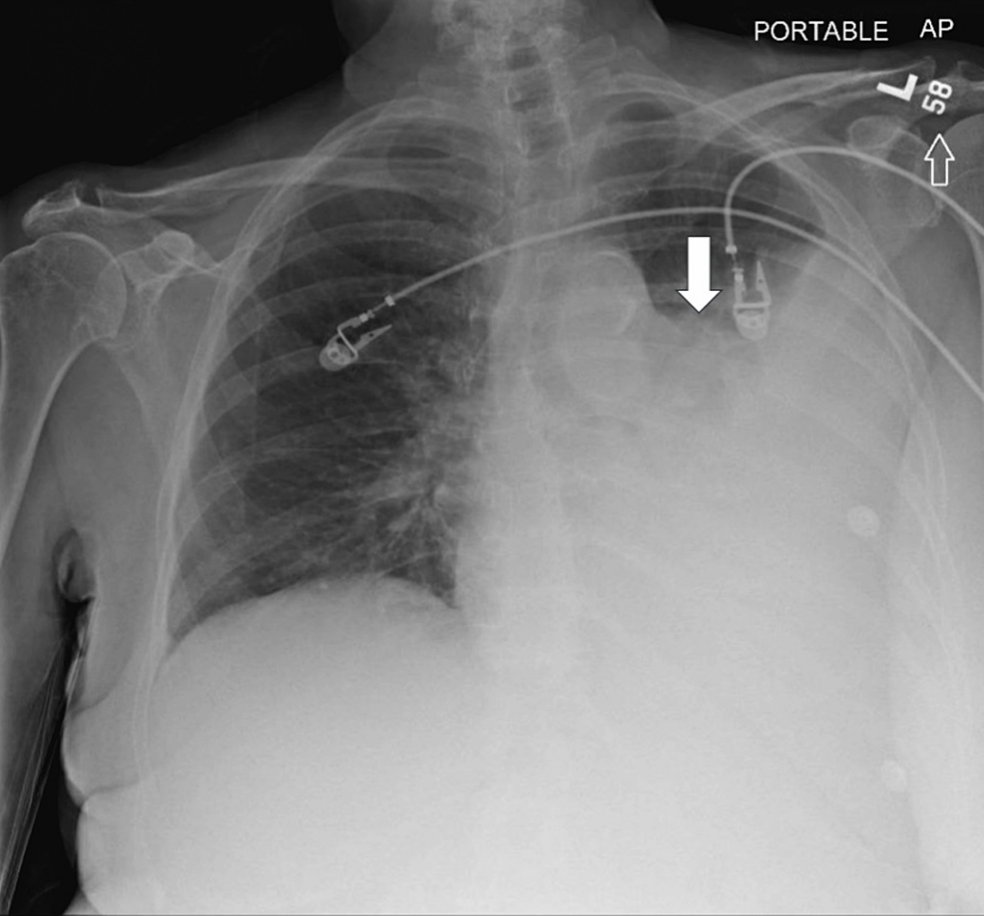
Chest x-ray of large pleural effusion (white arrow)

**Figure 2:**
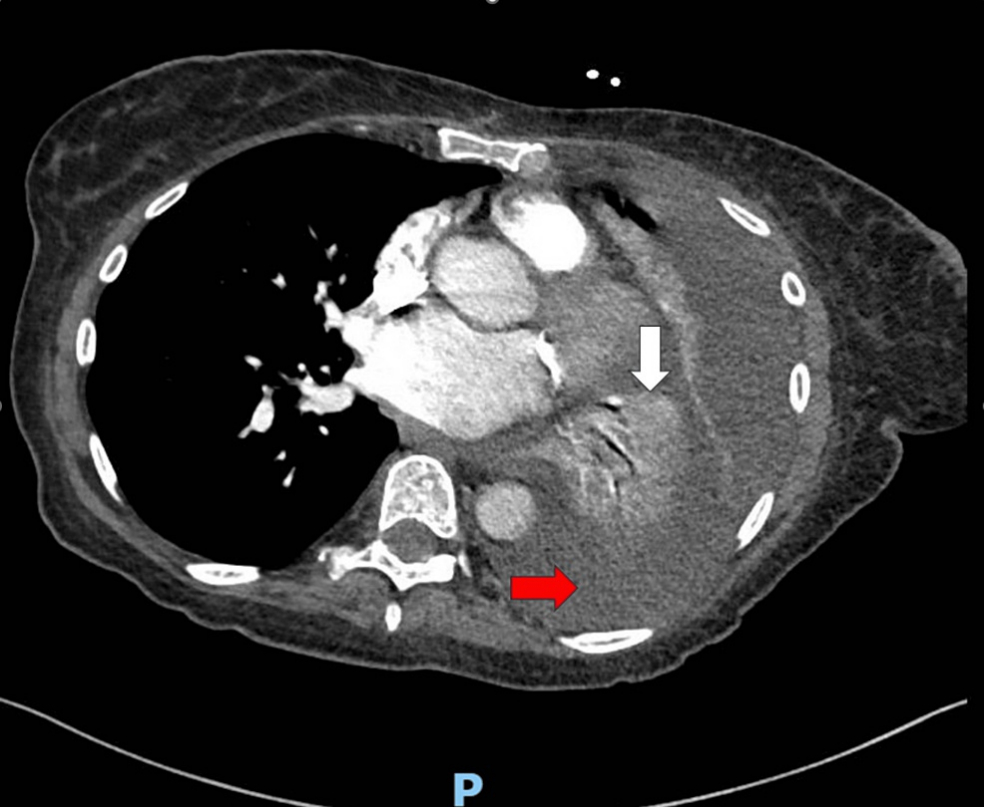
CT scan of the chest (mediastinal window) showing left pleural effusion (red arrow) with collapse of the left lower lobe (white arrow)

The patient was hemodynamically stable with blood pressure in the 130s systolic range, heart rate in the 80s range, and oxygen saturation in the mid-90s, on room air. Complete blood count showed a hemoglobin of 10.4 g/dL and white blood cell count (WBC) of 5.4 k/cumm. Complete metabolic panel was significant for a sodium of 123 mmol/L, and a potassium of 3.3 mmol/L. Additional lab work was significant for the following: magnesium of 1.6 mg/dL, and plasma osmolality of 248 mosm/k. A portable chest x-ray demonstrated a large left pleural effusion with basilar atelectasis (Figure 1). CT angiogram of the chest was negative for pulmonary embolism but demonstrated a large left pleural effusion with associated complete atelectasis of the left lower lobe and partial atelectasis of the left upper lobe (Figure 2). Additionally, there was mild enlargement of pre-tracheal mediastinal lymph nodes and no hilar lymphadenopathy.

The patient was admitted to the general medical floor, and a thoracentesis was completed with 1,660 cc of straw-colored pleural fluid removed. Pleural fluid chemistries revealed an exudative effusion with the following values: 1215 WBCs (with a lymphocytic predominance of 62%), lactate dehydrogenase (LDH) was 107 µ/L (serum LDH 166, reference range 140-271), and protein was less than 3.0 g/dL (serum protein was 4.6 g/dL). Bacterial, fungal, and acid-fast bacterial cultures of the pleural fluid were negative. Cytology was negative for malignant cells. An echocardiogram demonstrated a left ventricular ejection fraction of 55%-60%. The patient was placed on a fluid restriction as initial urine osmolality was 498 mosm/k, and urine sodium was 23 mmol/L. The patient’s hyponatremia improved with fluid restriction, increased protein intake, and the addition of sodium chloride tablets 1 g twice daily. The patient’s serum sodium improved to 132 mmol/L on the day of discharge (day 4 of the hospital stay).

Four days after discharge, the patient developed recurrent dyspnea with exertion and presented to the ED again as she was noted to be hypoxic with a saturation of 86% on room air at her primary care provider’s office. In the ED, a chest x-ray demonstrated a recurrent pleural effusion, and she subsequently underwent a second thoracentesis, removing 1,450 cc of pleural fluid. No fluid studies were ordered, and the patient was discharged home. 

She followed up in the pulmonary clinic 12 days after her initial presentation. Chest x-ray demonstrated re-accumulation of pleural fluid and she underwent another therapeutic thoracentesis in the office. Pleural fluid analysis was again exudative (WBC 630 with a 69% lymphocyte predominance, LDH 123, protein less than 3). Follow-up bacterial, fungal, acid-fast bacterial cultures and cytology of the pleural fluid were negative. The patient was referred to cardiothoracic surgery for video-assisted thoracoscopy, pleurodesis, and chest tube placement. During the procedure, 1,800 cc of blood-tinged fluid was drained and nodularity was noted in the left lateral aspect of the parietal pleura and diaphragm. Biopsy of both the pleura and diaphragm were positive for MCL. Follow-up PET demonstrated hypermetabolic lymphadenopathy in the neck, chest, abdomen, and pelvis. Additional hypermetabolism was noted with mass-like thickening of the rectum. Bone marrow biopsy demonstrated MCL. The patient was established with oncology and was initiated chemotherapy on bendamustine and rituximab.

The patient completed six cycles of bendamustine and rituximab and then was transitioned to rituximab only as maintenance therapy. A follow-up PET scan eight months after the initial demonstration demonstrated the resolution of adenopathy in her neck, chest, and abdomen with only faint residual activity from the mass involving her rectum. Given the reduced activity in the rectum, it is believed the original scan findings are related to the patient's MCL. Referral to colorectal surgery for colonoscopy is pending.

## Discussion

A literature search for case reports of MCL was conducted in January of 2023. Search terms were the following: Mantle Cell Lymphoma, primary pulmonary lymphoma, lymphoma of the pleura, B-cell mantle cell lymphoma, malignant pleural effusions, non-Hodgkin's lymphoma of the lung, extranodal lymphoma. The search directory utilized was PubMed. Cases that described synchronous neoplasms, which included MCL, and some other primary neoplasms were excluded. Presenting symptoms, the method used to diagnose MCL, duration of time required to diagnosis, and outcome, if presented, were organized into Table 1.

**Table 1:** Literature review of MCL MCL: mantle cell lymphoma, CT: computed tomography, VATS: video-assisted thoracoscopic surgery, NA: not available

Author	N	Presentation	Diagnosis	Modality of Diagnosis	Time to Diagnosis	Outcome
Papaioannou & Kostikas, 2005 [7]	1	Right pleural effusion, thrombocytopenia	MCL	VATS negative diagnosis made by mediastinoscopy	2 Months	NA
Woolery & Buttars, 2019 [8]	1	Fatigue and weight loss, pleural effusion identified on CT imaging	MCL	Bone marrow biopsy and pleural fluid cytology followed by thoracentesis	Same admission	NA
Kosaka & Koizumi, 2011 [9]	2	Right hilar lymphadenopathy (Case 1) and right pleural thickness (Case 2)	MCL	Case 1: Endobronchial ultrasound guidance transbronchial needle aspiration Case 2: Percutaneous chest wall biopsy	Same admission	Case 1: alive at 4 years Case 2: alive at 12 weeks
Shah & Dsilva, 2021 [10]	1	Left pleural effusion	MCL	CT-guided biopsy	Same admission	1-month follow-up
Köhl & Bardaro, 2022 [11]	1	Dyspnea	MCL	Bronchoscopy	Same admission	NA
Ding & Tang, 2022 [12]	1	Cough, 2-month duration	MCL	Bronchoscopy	Same admission	Referred to Oncology
Tong & Gan, 2018 [13]	1	2-year history of productive cough and worsening dyspnea, CT scan revealed hilar and mediastinal lymphadenopathy	MCL	Endobronchial biopsy via bronchoscopy	Same admission	NA
Anai & Hashisako, 2012 [14]	1	Previous diagnosis of MCL status-post treatment, presented with right pleural effusion	MCL	Thoracoscopy	NA	NA
Verde & Mcgeehan, 2008 [15]	1	20 lbs unintentional weight loss and night sweats. splenomegaly on CT scan	MCL, recurrent	Bone marrow biopsy	Same admission	2001 and 2008

Pleural effusions can complicate the course of lymphomas and can arise in the form of malignant pleural effusion or because of non-neoplastic complications. Although they can present at any time during the disease course, most commonly, they are identified at presentation. In a meta-analysis looking at pleural effusions in hematological malignancies by Fantin et al. [16], out of 1,216 cases, pleural effusions were most frequent in NHL (430 cases, 34.8%). In the same study, about 62% of patients had effusion at the time of diagnosis and almost all cases (174/176, 98.8%) had exudative effusions.

Patients with a pulmonary lymphoma present with a wide array of clinical manifestations from being asymptomatic to toxic appearing. In this case, the patient presented with vague symptoms of chest pain and palpitations, but upon a more detailed interview, the patient reported night sweats, unintentional weight loss, and generalized fatigue [17]. One exceeding rare extranodal site that has been found as an extranodal site is the pleura of the lung [5].

Thoracentesis provides a minimally invasive procedure that provides an avenue for quick tissue diagnosis, that may be done at the bedside. In expert hands, the complication rate is low and well tolerated by patients [18]. Endobronchial ultrasound-guided transbronchial needle aspiration is another option. This procedure is less invasive and may be utilized in patients who are not surgical candidates [19]. VATS, on the other hand, is associated with higher diagnostic yield, especially in the setting of pleural effusions of unknown etiology. Direct visualization allows for a more directed approach to obtaining tissue samples [20,21]. Yields can be as high as 97% via VATS procedures in exudative pleural effusions of unknown etiologies [21]. However, VATS is not without risk, complications like atrial fibrillation, pneumonia, and pain, are not uncommon post-operative complications in patients [22].

Pleural fluid cytology has a wide range of sensitivity depending on the tumor type and origin. The sensitivity of initial thoracentesis for malignant pleural effusions ranges between 40% and 60% [23]. In a recent meta-analysis, including 36 studies and about 6,057 patients, the overall diagnostic sensitivity of pleural fluid cytology for malignant pleural effusion was 58.2% [24]. Arnold et al. [25] reported sensitivity for cytological fluid analysis sampled via thoracentesis for adenocarcinoma was 79% versus hematological malignancies around 40%. In the case of Loveland et al. [26], the sensitivity of fluid cytology for lymphoma was as low as 20%.

## Conclusions

Pulmonary lymphomas are a rare entity. Pleural fluid cytology can be negative and should not be used to rule out malignancy. Clinicians must maintain a high index of suspicion when repeated thoracentesis fails to provide a diagnosis. Ultimately surgical procedures, like VATS, may be needed for definite diagnosis. Whether an aggressive approach with VATS should be pursued at the initial presentation of an exudative, lymphocyte-predominant pleural effusion, is still a topic of debate. A delay in diagnosis can result in a delay of care.
